# One household, two worlds: Differences of perception towards child marriage among adolescent children and adults in Indonesia

**DOI:** 10.1016/j.lanwpc.2021.100103

**Published:** 2021-02-05

**Authors:** Heribertus Rinto Wibowo, Muliani Ratnaningsih, Nicholas J Goodwin, Derry Fahrizal Ulum, Emilie Minnick

**Affiliations:** a Tulodo Indonesia, Indonesia; b UNICEF Indonesia, Indonesia

**Keywords:** Child marriage, Adolescents, Parents, Reproductive health, Indonesia

## Abstract

**Background:**

Indonesia is eighth in the world in the incidence of child marriage, with South Sulawesi province having one of the highest burdens (12.1%) in the country. The study explored the determinants of child marriage in Bone, South Sulawesi, in particular the differences between adolescents and parents.

**Methods:**

This is a cross-sectional study using a quantitative survey. A total of 1,004 respondents participated (500 parents and 504 adolescents aged 13-15 years).

**Findings:**

This study found that around one out of four parents or adolescents had perceptions that support determinants of child marriage.  A total of 25.8% of parents and 26.0% of adolescents agreed that a girl is ready for marriage once she starts menstruation. 25.6% of parents and 32.6% of adolescents agreed that girls aged over 18 who are not married are a burden to their families. Using the U-Mann Whitney , Kendall’s tau-b, and Pearson’s correlation test, these differences were found to be significant.

**Interpretation:**

Overall, the perceptions of parents and their adolescent children do not greatly differ, with some notable exceptions. Positive perceptions towards the benefits of child marriage are still prevalent among both parents and adolescents. This indicates that social norms supporting child marriage are still strong among these groups. Comprehensive interventions are needed to promote the benefits of marrying later, based on local cultural contexts and evidence on efficacy.

**Research in Context Panel**

**Evidence before this study**

The prevalence of child marriage in Indonesia is still high (10.82%). A large proportion of females are still entering into child marriage placing young mothers and their adolescents at significant risk. Several studies explore factors underlying child marriage practices include limited economic and educational opportunities, cultural norms, poverty, and parental concerns with safeguarding their daughters’ virginity. Parents are primary decision-makers in child marriage. The lack of power in decision making among girls also means parents tend to make decisions for their daughters about when and whom to marry but they do not fully bear the impact of those decisions.

**Added value of this study**

This study adds value as it is the first in Indonesia to explore perceptions among parents and adolescents related to the determinants of child marriage, e.g., socio-economic, education, and culture. This study is one of the first to document an intervention that links child marriage with menstrual health in Indonesia. The research also adds value to studies of making decision processes within families and communities, particularly on health and social issues.

**Implications of all the available evidence**

Previous evidence has established an understanding of the determinants of, and pathways to, child marriage in various contexts. Some of these examine the attitudes of parents and young people, few compare statistically significant cohorts of parents and their adolescent children in the same area, with none from Indonesia. Given the primacy of this relationship, our study explores perceptions of child marriage and its determinants between adolescents and parents in Bone, South Sulawesi. By exploring the differences and similarities between these two groups, we have produced findings that are being utilised to design tailored interventions and services to prevent child marriage.

Alt-text: Unlabelled box

## 1 Introduction

Child marriage and menstrual health are two significant and interconnected health and human rights concerns in many low- and middle-income countries, including Indonesia. Around 45% of Indonesian girls feel unprepared for their first period, and only 16% of adolescent girls and 9% of adolescent boys know that a woman's fertile period is halfway between her periods [1]. The child marriage burden remains too high in Indonesia, especially among female adolescents, despite positive long-term trends. Since 2008, there was a 3.85% decrease in the prevalence of child marriage from 14.67% in 2008 to 10.82% in 2019 [2,3]. The decline occurred in around three-quarters of provinces, mostly in rural areas. This continues a longer-term trend in the decline of child marriage in Indonesia - the risk of marrying before age 18 is less than half of what it was three decades ago. Based on 2017 data from the Village Data System (*Sistem Data Desa Kelurahan* = SDDK), from the 266,378 children in Bone District, the number of child marriage cases reached 2,635 cases [2].

A study in Indonesia targeting women aged 20-24 years found that child marriage has a socio-economic and health-related impact [2,4]. Women who were married under 18 years (13.8%) were more likely to live in poverty than women who were married above 18 years (10.1%). They also (58.9%) were more likely to have higher living expenses than those married above aged 18 years (52.8%).  For the health impact of child marriage, teenage pregnancy is correlated with maternal mortality and morbidity. Girls aged 10–14 years had five times the risk of dying during pregnancy or childbirth compared to 20-24 years, while this risk doubled in 15-19 years. The mortality rate for mothers under the age of 16 in middle and low-income countries is even sixfold higher. In Indonesia, the percentage of underweight babies born to women who were married below the age of 18 years was slightly higher (14.9%) than those born to women who married above 18 years (13.6%). Those married after the age of 18 years (81.8%) are more likely to give birth to babies weighing over 2.5 kg compared to those who are married under 18 years of age (76.3%). In 2015, a UNICEF study discussed the impact of girls’ limited ability to manage menstruation in schools, such as reduced participation and performance, absenteeism, and health risks. In 2015, a UNICEF study discussed the impact of girls’ limited ability to manage menstruation in schools, such as reduced participation and performance, absenteeism, and health risks [2,4].

Child marriage has a prominent place in the Sustainable Development Goals (SDGs), which has placed emphasis on gender equality and empowerment of women and girls. Target 5.3 aims to end harmful practices against girls and women, including child marriage. According to the Convention on the Rights of the Child (CRC), child marriage is defined as the marriage of a child younger than 18 years old. According to the Convention on the Rights of the Child (CRC), child marriage is defined as the marriage of a child younger than 18 years old [5]. The child marriage rate is defined as the percentage of ever-married women in Indonesia aged 20 to 24, married before age 18 (calculated by dividing the number of women of age 20-24 years who were married before the age of 18 by the total number of women aged 20-24 years). The global burden of child marriage is defined as the number of girls under age 18 who have already married plus the number of adult women who were married before age 18. Globally, one in six adolescent girls between the ages of 15 and 19 years is married or in a union, and as many as 700 million women were married as child brides in 2014 [6]. Gender norms, cultural practices, and economic challenges may be factor that drive child marriage. UNICEF data ranks Indonesia eighth in the world and second highest in Southeast Asia in terms of the percentage of marriages in which at least one of the spouses is under 18 years.

One study found that countries and societies with high gender inequality (e.g. laws and customs that exclude girls from decision-making or economic and political rights) are more likely to feature a high prevalence of child marriage [7]. Girls who marry before 18 years of age have increased risks of school dropout, gender-based violence, social isolation, poverty, non-use of contraception, high fertility, short birth intervals, unintended pregnancy, reproductive morbidities including obstetric fistula, maternal mortality, poor mental health, sexually-transmitted infections, and HIV/AIDS. Adolescents of adolescent mothers also face an increased risk of prematurity, malnutrition, and mortality [4]. Another study reported that early marriage reduces the chances of the woman completing her education and thereby minimising her chances of earning a decent livelihood. This leads to a vicious cycle where poverty and low literacy levels are coupled with religious, social, and cultural influences which lead to a higher prevalence of early marriages [8].

Marriage in Indonesia is regulated by Marriage Law No.1 of 1974. According to section 7, marriage is legal if the female is already aged 16 years (19 years for men). However, the law also allows marriages to be considered legal if they are carried out according to religious belief, known as *nikah siri’* (secret marriage). In September 2019, Law No. 16 was issued to amend the Marriage Law. The new law raised the age that girls can be married with parental permission from 16 to 19 years, in line with the age for boys. Marriages recognised by religious courts or officials are often later officially recognised by the government. Many people are also satisfied with merely fulfilling religious or traditional requirements for marriage. A study found that a large proportion of females are still entering into child marriage and cohabitation situations in Indonesia, placing young mothers and their adolescents at significant risk [9].

Inequality between women and men and gender-based violence remain serious concerns in Indonesia. To help address this situation, a program called “BERANI,” or Better Reproductive Health and Rights for All in Indonesia was launched by UNFPA and UNICEF in partnership with the Indonesian National Development Planning Agency (Badan Pembangunan dan Perencanaan Nasional/Bappenas) and the Government of Canada. The name derives from the Indonesian word *berani,* which means courageous. One of BERANI's aims is to prevent child marriage and improve menstrual hygiene management. Bone District, South Sulawesi, was selected as the site for a pilot project and a baseline study was conducted in 2019. The baseline study found seven main contributing factors to child marriage in Bone, presented below in order from strongest to weakest. The first is the *siri’* (shame) felt by parents around their daughter's unwanted pregnancy. Second, and related, is the parents’ determination to avoid the fear of their adolescent dating, which is associated with sexual relations and the risk of unwanted pregnancy - and therefore *siri’*. A third factor is an obedience and dependence of (rural) children. The fourth determinant is economic, when parents can no longer support their children and believe marrying them will reduce this burden, often also felt by the adolescents. Related to this is the fifth determinant, which is education, as adolescents who drop out of school are more likely to marry younger. A sixth determinant is the role of media as a channel used to connect young people and promote the benefits of marriage. The seventh and final determinant is the limited knowledge of the negative impacts of child marriage [10]. Lack of communication on health and relationships between parents and adolescents may push young people to seek other information sources, such as peers and social media, that may be unreliable or inaccurate [11].

Child marriage and menstrual hygiene issues are connected. In some countries, parents and communities view a girl's first menstrual period as a sign that she is a woman and ready for marriage and motherhood. Some child marriages also happen because the girl gets pregnant. One reason she becomes pregnant is that the couple (mostly the boy) didn't know she had her period, or what it means. And they didn't know they can become pregnant by having sex one time. Menstrual health management (MHM) is one part of sexual and reproductive health (SRH) that is essential to enable young women to manage their well-being and reproductive choices.

The relationship between parent and adolescent is fundamental to shaping children's trajectories through adolescence and provides the considerable potential to improve youth sexual and reproductive health (SRH). This study explores child marriage-related perceptions among parents and adolescents particularly perceptions related to the determinants of child marriage (e.g., socioeconomic factors, culture) including the perception of whether a girl is ready for marriage once she starts menstruating. This study also explores whether parents and adolescents have different perceptions of child marriage. Based on some studies, we found that parents’ decision contributes to child marriage. By exploring the differences between these two groups, it could be utilised to design interventions and communication strategy for child marriage prevention. As we may need different approaches for parents and adolescents in the child marriage campaign. The study was conducted as a guidance to develop a child marriage prevention strategy consisting of a theory of change, monitoring and evaluation framework, behaviour change approach, and communication materials for implementation as part of the BERANI pilot program in Bone, South Sulawesi.

## 2 Methods

### 2.1 Study design and participants

This study used a cross-sectional design, with researchers making observations or measurements of variables at a certain time. The participants consisted of two groups: parents or caregivers in households with children aged 13-15 years and adolescents aged 13-15 years. The study was conducted in Bone District, one of 24 districts in South Sulawesi, located to the east of the capital city of the South Sulawesi Province. The total area of 4,559 km^2^ is divided into 27 districts/kecamatan with 372 villages/kelurahan. The population of Bone Regency in 2018 was 869,016 people spread across 27 sub-districts. The total population in Bone age under 18 years was 266,737 people. According to data from the Ministry of Education, the total adolescents aged 13-15 years in Bone in 2018 was 40,621 children. The study was conducted in Bone as Bone is part of South Sulawesi with the prevalence of child marriage in South Sulawesi was 14.1% higher than the average of child marriage prevalence at the national level (11.2%) in 2018, and in 2019, the prevalence was 12.1%, still higher than the national average 10.82%.

### 2.2 Procedures

#### 2.2.1 Adolescent survey sampling

As we compared the differences between boys and girls, the minimum number of participants for each group was calculated using Hulley et al's 2013 method, where N (sample) = 16: (standardised size effect)^2^. The standardised size effect to be used is 0.3, with a confidence interval of 95%, and power (β) of 80%. Using this formula, the sample for each group needed was 177 people. The total required sample for both boys and girls were 354 adolescents aged 13-15 years. Adding 10% to allow for problems, the total sample recruited was 389 adolescents, both in and out of school. Samples were taken from 12 intervention schools in 6 sub-districts and 4 schools from control areas. We recruited 30 respondents in each school. For the out of schools, we recruited 24 adolescents aged 13-15 years using a purposive sampling method. The intervention areas were six villages selected by UNICEF as the implementing areas for the BERANI program, while the control areas were the areas that did not receive the BERANI program in Bone District, South Sulawesi, Indonesia. For samples selected within the school, the researcher used simple random sampling from a list of students aged 13-15 years. For samples selected outside of school, the researcher used purposive sampling, with the following criteria: (1) male/female adolescents aged 13-15 years and (2) dropping out of school/not continuing their education.

#### 2.2.2 Parent/caregiver survey sampling

The same sampling method and size for the adolescent survey was used for the parent/caregiver survey, with a total sample recruited needed of 389 people. Respondents aged 30 years and over from 8 villages in Bone who have adolescents aged 13-15 years were recruited. With the help of the village government, we mapped the households and determined which respondents were visited by using random techniques. We select one house for every 10 houses, and if there were no families with adolescents aged 13-15 years within the 10 houses range, we visited the house next door until we found those with children aged 13-15 years, ask the households to join the study, and continued the data collection.

#### 2.2.3 Outcomes

This study will show different perceptions among parents and adolescents toward child marriage, its determinants, and its impact. The independent variables were demographics: gender, type of respondents (parent/caregiver vs adolescent), and location. The dependent variables in this study were the level of perception related to child marriage and menstrual health. This study also explored factors that influence parent/caregiver intention to arrange a marriage for their adolescents. This included adolescent intention and acceptance to marry early, as well as parent/caregiver efforts to provide education on health and relationships.

#### 2.2.4 Data collection

For the quantitative study, survey-style interviews of parents/caregivers were conducted by enumerators. There were eight enumerators with experience in data collection and various educational backgrounds, some had graduated from universities and some were university students. For adolescents at school, data was collected through questionnaires. The questionnaire used paper, whilst for the parent/caregiver survey, data collectors used the mWater Surveyor App on tablets. The survey took approximately 50–60 minutes to complete. Questionnaires were developed based on references from several standard questionnaires with modification of questions to focus more on the practices of adolescents and parents/caregivers around child marriage and menstrual health issues. Data collection was carried out by the main researcher and assisted by eight enumerators. Before data collection, training for the enumerators and pilot testing was conducted. The training aimed to ensure the uniformity of the data collection and so the enumerators followed the research protocol. The pilot test was conducted to analyze the level of understanding of the participants of the survey questions and to improve the interviews. Pilot testing with thirty selected households and thirty students was conducted before the data collection. Ethical Clearance was obtained from the University of Indonesia's Center for Health Research (No.256/UN2.F10/PPM.00.02/2019).

#### 2.2.5 Statistical analysis

SPSS 22.0 for PC was used to analyze quantitative data. To analyze demographic data, such as gender, the use of media, and technology, descriptive statistics were used. To answer the research question, inferential statistics were used. The questions of attitudes related to child marriage were explored as the dependent variables in this study were taken from the Child Marriage Acceptability Index (CMAI) developed by Plan International and standard questionnaire from UNICEF related to child marriage. The responses were arranged along a 7-level Likert scale consisting of: strongly disagree (1), disagree (2), slightly disagree (3), neither disagree nor agree (4), slightly agree (5), agree (6), and strongly agree (7). For further analyses, we classified the responses above into two categories: (1) agree category (strongly agree/agree/slightly agree) and (2) disagree category (strongly disagree/disagree/slightly disagree). As the nature of data is ordinal, we used the U-Mann Whitney Test to analyze the differences in perception between parents/caregivers and adolescents. To analyze the association between attitudes and gender, type of respondents, and locations, we used Kendall's tau-b for Likert-type items and Pearson's correlation test for Likert scales.

#### 2.2.6 Role of the funding source

UNICEF and UNFPA in partnership with the Indonesian Government, and the Canadian Government launched the BERANI (Better Reproductive Health and Rights for All in Indonesia) in 2018. One of the components in the BERANI program is the baseline study in Bone. This study is part of the baseline study, as we explore further the findings to address the research questions. The funding used to manage the study including for the data collection with enumerators and data analysis.

## 3 Results

### 3.1 Sample characteristics from parents/caregivers

A total of 500 respondents joined the parents/caregivers survey, chosen randomly in 6 intervention areas (Ajangngale, Awangpone, Cina, Salomekko, Tellusiattingnge and Ulaweng) and two control subdistricts (Libureng and Bontocani). As shown in Table 1, 64.4% (n=322) were from intervention areas while 35.6% (n=178) were from the control. 416 respondents were female (83.2%), while 84 were male (16.8%). Most respondents were mothers or primary caregivers of adolescents-aged 13-15 years (72.2%, n=361) whilst 20% (n=100) were heads of households. Out of 361 primary caregivers, 86.3% (n=359) were female respondents whilst 2.4% (n=2) were male. 38.4% (n=192) had completed elementary school, 21.4% (n=107) completed junior high school, and 17% (n=85) graduated from senior high school. For heads of household, 41% (n=205) completed elementary school, 17.4% (n=87) graduated from junior high school and 16.2% (n=81) graduated from senior high school.

**Table 1:** Sample characteristics of households.

Sample Characteristics	n	%
**Sub-district**		
Ajangngale	54	10.8
Awangpone	58	11.6
Bontocani	62	12.4
Cina	52	10.4
Libureng	116	23.2
Salomekko	32	6.4
Tellusiattingnge	78	15.6
Ulaweng	48	9.6
**Gender**		
Male	84	16.8
Female	416	83.2
**Education of parents/caregivers**		
No school/out of school	15	3.0
Graduated from Madrasah Ibtidaiyah (MI)	1	0.1
Graduated from Madrasah Tsanawiyah (MTs)	14	2.8
Graduated from Madrasah Aliyah (MA)	5	1.0
Graduated from Elementary School	192	38.4
Graduated from Junior High School	107	21.4
Graduated from High School	85	17.0
Not complete Madrasah Ibtidaiyah (MI)	1	0.2
Not complete Madrasah Tsanawiyah (MTs)	1	0.2
Not complete Madrasah Aliyah (MA)	0	0
Not complete Elementary School (SD)	31	6.2
Not complete Junior High School (SMP)	11	2.2
Not complete High School	8	1.6
University or College	29	5.8
**Status of parents in the household**		
Mother/main caregiver	361	72.2
Head of the family	100	20.0
Another adult male	1	0.2
Other adult women/child guardians	38	7.6

### 3.2 Sample characteristics from adolescents

A total of 504 adolescents aged 13-15 years joined the study, consisting of 24 out-of-school and 480 students from 16 schools in 8 sub-districts in Bone (6 intervention sub-districts and 2 control). As shown in Table 2, there were 254 boys (50.4%), and 250 girls (49.6%). Most respondents were in grade IX (224 respondents, 44.4%), the rest were in grade VII and VIII, with most (38.1%) aged 13 years (n=192).

**Table 2:** Sample characteristics of adolescents.

Sample Characteristics	n	%
**Sub-district**		
Ajangngale	63	12.5
Awangpone	63	12.5
Bontocani	63	12.5
Cina	63	12.5
Libureng	63	12.5
Salomekko	63	12.5
Tellusiattingnge	63	12.5
Ulaweng	63	12.5
**Gender**		
Boys	254	50.4
Girls	250	49.6
**Grade of schools**		
Grade VII	80	15.9
Grade VIII	176	34.9
Grade IX	224	44.4
Not schools/out of schools	24	4.8

Based on Table 3, several statements related to child marriage perception differ between parents and adolescents. 25.8% of parents and 26.0% of adolescents agreed that a girl is ready for marriage once she starts menstruating. 29.6% of parents and 33.4% of adolescents agreed that marrying girls can help protect family honours/reputation. 29.2% of parents and 33.0% of adolescents agreed that marrying boys can help protect family honours/reputation. 23.0% of parents and 24.6% of adolescents agreed that girls who give birth between 15-18 years are more likely to have a healthy pregnancy/ baby (compared to girls over 18). 23.2% of parents and 26% of adolescents agreed that marrying young girls can help resolve financial problems in the family. 23.2% of parents and 27.4% of adolescents agreed that marrying young boys can help resolve financial problems in the family. 25.2% of parents and 29.4% of adolescents agreed that early marriage of girls can help prevent sexual violence, assault, and harassment.

**Table 3:** Perception of child marriage for adolescent children and adults (n=1004).

Statements	STA		A		SLA		N		SLD		D		STD	
	n	%	n	%	n	%	n	%	n	%	n	%	n	%
*A girl is ready for marriage once she starts menstruating.*														
Parents	9	1.8	106	21.2	14	2.8	31	6.2	33	6.6	301	60.2	4	0.8
Adolescents	25	5.0	33	6.5	73	14.5	44	8.7	53	10.5	192	38.1	53	10.5
*Marrying girls can help protect family honours/reputation.*														
Parents	10	2.0	115	23.0	23	4.6	38	7.6	48	9.6	261	52.2	5	1.0
Adolescents	30	6.0	64	12.7	74	14.7	55	10.9	63	12.5	154	30.6	64	12.7
*Marrying boys can help protect family honours/reputation.*														
Parents	8	1.6	115	23.0	23	4.6	40	8.0	43	8.6	267	53.4	4	0.8
Adolescents	27	5.4	68	13.5	71	14.1	59	11.7	56	11.1	165	32.7	58	11.5
*Girls who give birth between 15-18 years are more likely to have a healthy pregnancy/ baby (compared to girls over 18).*														
Parents	3	0.6	93	18.6	19	3.8	58	11.6	45	9.0	272	54.4	10	2.0
Adolescents	16	3.2	42	8.3	66	13.1	70	13.9	78	15.5	173	34.3	78	15.5
*Marrying young girls can help resolve financial problems in the family.*														
Parents	9	1.8	81	16.2	26	5.2	32	6.4	55	11.0	289	57.8	8	1.6
Adolescents	9	1.8	49	9.7	73	14.5	67	13.3	79	15.7	174	34.5	79	15.7
*Marrying young boys can help resolve financial problems in the family.*														
Parents	6	1.2	79	15.8	31	6.2	31	6.2	53	10.6	292	58.4	8	1.6
Adolescents	22	4.4	47	9.3	69	13.7	75	14.9	53	10.5	169	33.5	69	13.7
*Early marriage of girls can help prevent sexual violence, assault, and harassment.*														
Parents	4	0.8	86	17.2	36	7.2	45	9.0	72	14.4	246	49.2	11	2.2
Adolescents	15	3.0	55	10.9	78	15.5	67	13.3	64	12.7	155	30.8	70	13.9
*Early marriage of boys can help prevent sexual violence, assault, and harassment.*														
Parents	7	1.4	102	20.4	32	6.4	43	8.6	70	14.0	238	47.6	8	1.6
Adolescents	21	4.2	59	11.7	78	15.5	62	12.3	68	13.5	158	31.3	58	11.5
*Physical changes in appearance is a sign that a girl is ready for marriage.*														
Parents	10	2.0	93	18.6	27	5.4	45	9.0	51	10.2	267	53.4	7	1.4
Adolescents	14	2.8	55	10.9	65	12.9	95	18.8	65	12.9	148	29.4	52	10.3
*Girls over 18 who are not married are a burden to their families.*														
Parents	14	2.8	97	19.4	17	3.4	28	5.6	44	8.8	294	58.8	6	1.2
Adolescents	22	4.4	61	12.1	81	16.1	81	16.1	64	12.7	139	27.6	56	11.1
*Boys over 18 who are not married are a burden to their families.*														
Parents	10	2.0	102	20.4	19	3.8	25	5.0	48	9.6	289	57.8	7	1.4
Adolescents	25	5.0	54	10.7	70	13.9	87	17.3	66	13.1	149	19.6	53	10.5
*Most adolescent girls prefer to marry before 18.*														
Parents	9	1.8	54	10.8	7	1.4	41	8.2	50	10.0	328	65.6	11	2.2
Adolescents	18	3.6	48	9.5	65	12.9	70	13.9	58	11.5	156	31.0	89	17.7
*Parents expect adolescent girls to become married before the age of 18 years.*														
Parents	10	2.0	46	9.2	7	1.4	29	5.8	30	6.0	367	73.4	11	2.2
Adolescents	30	6.0	40	7.9	59	11.7	66	13.1	44	8.7	173	34.3	92	18.3
*Parents would look down on adolescent girls if they became pregnant before they became married.*														
Parents	34	6.8	219	43.8	36	7.2	30	6.0	35	7.0	142	28.4	4	0.8
Adolescents	84	13.7	114	22.6	87	17.3	69	13.7	30	6.0	80	15.9	40	7.9
*I will obey my parents if they ask me to marry even though I am still at school.*														
Parents	2	0.4	35	7.0	11	2.2	35	7.0	34	6.8	368	73.6	15	3.0
Adolescents	17	3.4	37	7.3	56	11.1	64	12.7	43	8.5	175	34.7	112	3.4

STA: Strongly Agree, A: Agree, SLA: Slightly Agree, N: Neither disagree nor agree (neutral).

SLD: Slightly Disagree, D: Disagree, STD: Strongly Disagree.

28.2% of parents and 31.4% of adolescents agreed that early marriage of boys can help prevent sexual violence, assault, and harassment. 26% of parents and 26.6% of adolescents agreed that physical changes in appearance are a sign that a girl is ready for marriage. 25.6% of parents and 32.6% of adolescents agreed that girls over 18 who are not married are a burden to their families. 26.2% of parents and 29.6% of adolescents agreed that boys over 18 who are not married are a burden to their families. 14.0% of parents and 26.0% of adolescents agreed that most adolescent girls prefer to marry before 18. 12.6% of parents and 25.6% of adolescents agreed that parents expect adolescent girls to marry before the age of 18 years. 57.8% of parents and 53.6% of adolescents agreed that parents would look down on adolescent girls if they became pregnant before they marry. 9.6% of parents and 21.8% of adolescents agreed that they will obey their parents if they asked them to marry even though still at school.

A Mann Whitney U-Test test results showed a statistically significant difference in terms of responses to the statement, “a girl is ready for marriage once she starts menstruating” between the type of respondents, p = 0.034, with a mean rank 520.69 for parents and 484.45 for adolescents (see Table 4). In terms of the statement, “Girls over 18 who are not married are a burden to their families”, there was a significant difference between the type of respondents, p = 0.002, with a mean rank of 475.71 for parents and 529.80 for adolescents. The other significant differences were found in terms of the statements, “Boys over 18 who are not married are a burden to their families” (p = 0.009), “Most adolescent girls prefer to marry before 18” (p=0.011), and “parents expect adolescent girls to get married before the age of 18 years” (p = 0.002).

**Table 4:** Analysis of difference using Mann Whitney U-test.

Statements	Type of Respondents (Mean Ranks)		U Value	p-Value
	Parents	Adolescents		
A girl is ready for marriage once she starts menstruating	520.69	484.45	116,905.000	0.034*
Marrying girls can help protect family honour/ reputation	500.42	504.57	127,041.000	0.813
Marrying boys can help protect family honour/ reputation.	498.94	506.03	127,778.000	0.685
Girls who give birth between 15-18 years are more likely to have a healthy pregnancy/ baby (compared to girls over 18).	511.56	493.51	121,467.500	0.300
Marrying young girls can help resolve financial problems in the family	506.06	498.97	124,220.500	0.682
Marrying young boys can help resolve financial problems in the family.	492.29	512.63	131,105.000	0.241
Early marriage of girls can help prevent sexual violence, assault, and harassment.	501.66	503.33	126,418.500	0.925
Early marriage of boys can help prevent sexual violence, assault, and harassment.	502.33	502.67	126,085.500	0.985
Physical changes in appearance is a sign that a girl is ready for marriage	487.42	517.46	133,541.000	0.087
Girls over 18 who are not married are a burden to their families	475.71	529.08	139,395.000	0.002*
Boys over 18 who are not married are a burden to their families.	479.56	525.26	137,470.500	0.009*
Most adolescent girls prefer to marry before 18.	480.63	524.20	136,935.000	0.011*
Parents expect adolescent girls to get married before the age of 18 years.	476.80	528.00	138,851.000	0.002*
Parents would look down on adolescent girls if they get pregnant before they get married	503.21	501.80	125,465.000	0.937
I will obey my parents if they ask me to marry even though I am still at school.	489.39	515.50	132,554.500	0.119

Based on Kendall's Tau-b test results, there was a statistically significant association in terms of the statement, “Most adolescent girls prefer to marry before 18 years by type of respondents between parents and adolescents (p=0.011; r=0.072) (see Table 5).

**Table 5:** Analysis of likert type association using Kendall's Tau-b test.

Statements	Type of respondents		Gender		Locations	
	Corr. Coefficient (CC)	p -Value	CC	p Value	CC	p Value
A girl is ready for marriage once she starts menstruating	−0.061	0.034	−0.355	0.000	−0.115	0.000
Girls who give birth between 15-18 years are more likely to have a healthy pregnancy/ baby (compared to girls over 18).	−0.030	0.300	−0.076	0.008	−0.101	0.703
Girls over 18 who are not married are a burden to their families	0.087	0.002	−0.018	0.532	−0.011	0.010
Boys over 18 who are not married are a burden to their families.	0.074	0.009	−0.087	0.002	−0.062	0.029
Most adolescent girls prefer to marry before 18.	0.072*	0.011	−0.074	0.009	−0.073	0.045
I will obey my parents if they ask me to marry even though I am still at school.	0.045	0.119	−0.130	0.000	−0.057	0.001

⁎ p-value <0.05 and r count > r table (r=0.062): significant.

Based on the Pearson's Correlation test, there was a statistically significant association in terms of responses to the statement, “physical changes in appearance is a sign that a girl is ready for marriage” by type of respondents between parents and adolescents (p=0.000; r=0.143). There was a statistically significant association in terms of the statement, “parents expect adolescent girls to get married before the age of 18 years” by gender between female and male (p=0.038; r=0.066); by location between intervention and control (p=0.000; r=0.119). There was a statistically significant association in terms of the statement “Parents would look down on adolescent girls if they get pregnant before they get married” by type of respondents between parents and adolescents (p=0.000; r=0.122) (see Table 6).

**Table 6:** Analysis of Likert scale association using Pearson's Correlation test.

Statements	Type of respondents		Gender		Locations	
	Pearson's Correlation	p-value	Pearson's Correlation	p-value	Pearson's Correlation	p-value
Marrying girls can help protect family honour/reputation	0.018	0.559	−0.043	0.170	−0.037	0.244
Marrying boys can help protect family honour/ reputation.	0.024	0.454	−0.060	0.058	−0.042	0.188
Marrying girl young can help resolve financial problems in the family	0.003	0.916	−0.048	0.126	−0.058	0.068
Marrying a boy young can help resolve financial problems in the family.	0.052	0.097	−0.096	0.002	−0.062	0.050
Early marriage of girls can help prevent sexual violence, assault, and harassment.	0.017	0.585	−0.043	0.172	−0.057	0.073
Early marriage of boys can help prevent sexual violence, assault, and harassment.	0.010	0.762	−0.002	0.937	−0.016	0.611
Physical changes in appearance is a sign that a girl is ready for marriage	0.143*	0.000	−0.104	0.001	−0.151	0.000
Parents expect adolescent girls to get married before the age of 18 years.	0.005	0.868	0.066*	0.038	0.119*	0.000
Parents would look down on adolescent girls if they get pregnant before they get married	0.122*	0.000	−0.155	0.000	−0.101	0.000

⁎ p-value <0.05 and r count > r table (r=0.062): significant.

## 4 Discussion

### 4.1 Support for child marriage still high because of pride

This study found that 29.6% of parents and 33.4% of adolescents strongly agreed/agreed/slightly agreed that marrying girls can help protect family honours /reputation. The perception of the benefits of child marriage in terms of protecting family honours /reputation was significantly higher among adolescents than those of parents. This is because the mind frames of adolescents that marriage will protect the honours of the family as future successors. So, when a marriage is not held even though it has been planned regardless of the age of the child, the marriage must still be conducted to protect the family's honours because it has been planned. In South Sulawesi, A study reported the practice of child marriage among Buginese and Makassarese to be socially and culturally supported. People often believe that the sooner women are married, the better their economic and financial position will be, as well as the family's honor [12]. However, when women marry late or stay single, it is said their prosperity and luck are ‘closed and late’ which means that it can bring shame to family honours. Often the community blame women as being ‘poor’ and have negative perceptions of them being unmarried. As most Buginese and Makassarese are Muslim, they often believe that child marriage is justifiable because there are no clear Islamic teachings either in the Quran or Hadith that dictate the minimum age of marriage, and it is merely on physical and biological aspects such as *aqil baligh* (coming of age). They tend to seek normative religious-based texts which eventually claim the truth based on Islamic teaching. For them, women and men can marry if they have already achieved *aqil baligh*, without considering their age [12].

### 4.2 Support for child marriage still high because of economic factors

Marriage is sometimes used as a means to rise out of poverty and into higher social status. Around 23.2% of parents and 26.0% of adolescents strongly agreed/agreed/slightly agreed that marrying a girl young can help resolve financial problems in the family; 25.6% of parents and 32.6% of adolescents strongly agreed/agreed/slightly agreed that girls over 18 who are not married are a burden to their families. Different perceptions related to economic problems can lead to stronger adolescent perceptions towards the benefits of child marriage than parents. Some adolescents see aspects of marriage as helping to improve the economic life of the married couple or child. The perception of raising the standard of living and solving economic problems carries more benefits than the negative consequences of marrying underage and illegally. The amount of dowry given, and debt incurred, can also contribute to child marriage.

One study found that people with low incomes in Vietnam tend to be younger than people in middle- and higher-income families [13]. A study in India reported that literacy and socio-economic status of the mother and her parents were a major determinant in deciding the age at marriage [8]. A study also reported that poverty and poor education are underlying causes of child marriage, which is usually against the will of girls who desire to be educated [14]. Moreover, findings from quantitative studies in Indonesia also revealed that marriage is sometimes used as a way out of poverty [15]. This indicates that women's education, work status before marriage, husband's education, and current residence are the predictors for early marriage in Java, with education as the strongest. Women who are married at a younger age also mostly live in poverty [14]. These negative effects will often also be experienced by their children and can continue in future generations. However, the Bureau of Statistics also reported the child's age at marriage is strongly associated with poverty, however, a high prevalence of child marriages was also found in provinces with low poverty levels. This might suggest that poverty is also used as the reason given for child marriage, but motivations are more complicated [2].

### 4.3 Child marriage and unwanted pregnancy

Around 57.8% of parents and 53.6% of adolescents strongly agreed/agreed/slightly agreed that parents would look down on adolescent girls if they became pregnant before they marry. There are differences in perception related to unwanted pregnancy, with stronger support for child marriage as a solution from parents than adolescents. Bugis culture encompasses a belief in the importance of ‘*siri’* (shame), which includes a strong social norm that it is shameful if a child is pregnant outside of marriage so she must be married even though she's underage. Parents believe this is preferable to the family bearing the shame. Child marriage is a relatively common phenomenon throughout Indonesia, with practices such as covering the shame of extra-marital sexual relations, manipulating age and wedding dates, falsifying marriage dispensation, as well as handling teenage pregnancy by arranging a marriage, are found throughout the country, including in non-Muslim areas [16].

One commonly hypothesised reason for child marriage in the United States is the so-called shotgun wedding, entered into under pressure from family members or others who aim to avoid perceived stigma resulting from premarital sexual activity and pregnancy [17]. A study in fifteen countries, including Indonesia, found that child marriage is associated, among other factors, with lower age at first birth, higher fertility, lower contraceptive use, and higher risks of unplanned pregnancies and pregnancy termination [18]. Children's unwanted pregnancy and early marriage are still quite high in Indonesia. The parent's role is an important factor in a child's unwanted pregnancy and early marriage. Permissive parents (those whose children married underage) had 2.18 times greater unwanted pregnancies compared to less permissive parents when adjusted for other variables. Of the permissive parents, 68.3% were from strongly religious families, and 25.0% believed the function of the family is a reproduction, which was significantly associated with unwanted pregnancies. A large number of unwanted pregnancies and early marriages are associated with parental permissiveness, religiosity, and belief in the reproductive function of families [19].

### 4.4 Menstruation, puberty and child marriage

Around 25.8% of parents and 26.0% of adolescents strongly agreed/agreed/slightly agreed that a girl is ready for marriage once she starts menstruating, 26.0% of parents and 26.6% of adolescents agreed to physical changes in appearance are a sign that a girl is ready for marriage. Perceptions about menstruation and puberty as a contributor to child marriage are stronger in adolescents than parents. This is due to observations by adolescents that children who legally are not ready to marry (aged under 19 years), actually can marry because the signs of puberty, especially menstruation, indicate their readiness. This perception towards physical change and menstruation was still strong among parents and adolescents.

Considering the practices of the Musgum people in Cameroon and Chad, participants in one study stressed the importance of physical preparedness for deciding whether a girl is ready for marriage. One older man, for instance, said: ‘*It is when a girl already has breasts that she can be sent for marriage*’. Another older man confirmed: ‘*Yes, she is 15 years old. So, she can get married normally. But if she has a small body and is without breasts, she can delay the wedding*’. One young man explained: “*Among the Musgum, what matters is when the girl has her first period. The girl may see her first menstruation when she's 13; then she can get married; age depends on the body of the girl*” [20].

A legal case in India featured a man who had advocated for lowering the marriageable age of girls, saying a man aged 30 years should be able to marry a 12 years old girl who “charms his heart”, and a man of 24 years should be able to marry a girl eight years old. He also said girls should be married before they reach puberty, if they marry after first menstruation, they become impure and parents are likely to go to hell. In fear of going to hell, parents started arranging marriage for their daughters before age ten, sometimes at four or five, and some even went to the extreme of getting them married in the cradle [21]. Another study from India reported that girls in their community have their first period between the ages of ten and twelve. For those living in the village, *streeachar* (feminine rituals) were performed to mark a girl's puberty and menarche. While these rituals have been changed or lost with modernisation, the onset of menstruation is considered a ‘warning’ signal to the girl's parents. As soon as she attains puberty, the elders begin to alert her parents to start looking for a groom, to protect her virginity and honours [22].

### 4.5 Child marriage and sexual harassment

Around 25.2% of parents and 29.4% of adolescents strongly agreed/agreed/slightly agreed that early marriage of girls can help prevent sexual violence, assault, and harassment. The perception of child marriage as protection against sexual harassment is stronger in adolescents than in parents. Fed by the media portrayal of the frequency and uncontrolled incidence of rape and other sexual violence, adolescents believe that it will happen to them. These adolescents believe that marriage, even underage, is better than dealing with continuous violence and harassment. By extension, girls attain sexual maturity, their vulnerability to sexual violence rises, along with parental apprehension about their safety. This is especially apparent in traditional and poor communities where girls’ roles are often expected to be that of mothers and wives. Parents who marry their children before they reach the legal age are typically motivated by social and sexual norms (often attaching low value to daughters), as well as poverty or humanitarian crises. Confronted with social pressure and family hardship, they may seek in marriage a form of protection to shield their children from destitution, household food insecurity, and, ultimately, sexual harassment [23].

However, the evidence shows girls who marry below 18 years are more likely to experience domestic violence than their peers who marry later. For example, in Peru, where more than half of women report having experienced physical or sexual violence, child marriage has been found to increase a woman's chances of being abused [24]. Girls who marry early are more likely to believe that a man is sometimes justified in beating his wife than women who marry later. For example, in Kenya, 36% of girls married before 18 believe that a man is sometimes justified in beating his wife, compared to 20% of married women [25]. Girls with low bargaining power in the household are more likely to experience violence by an intimate partner. Women with low levels of education and adolescents ages 15 to 19 are at higher risk of violence than better educated or older women [24]. Ending child marriage must be a priority in the global effort to end violence against women.

## 5 Conclusion

The prevalence of positive perceptions of the benefits of child marriage is still high among both parents and adolescents in Bone. However, the study found how they differed, for example, the perception of the benefits of child marriage towards family honour and reputation was stronger among adolescents than parents. The responses to the economic benefits of child marriage also highlight this difference in perception between parents and adolescents. For parents, who may be more sensitive to economic issues, marrying girls at a young age was seen as a way to resolve financial problems and avoiding the burden of those aged over 18 years who are not married. Marriage is still seen as a way out of poverty and pulls young people towards its promises of a better life. The power of shame around unwanted pregnancy contributes to the additional push to convince parents to seek marriage. Perceptions of a girl's readiness based on signs of puberty provide the behaviour trigger for action. Beliefs in the benefits of child marriage as a way to prevent sexual violence provide additional impetus, especially for fearful girls. Comprehensive interventions based on behavioural science and cultural understanding are needed to provide high-quality services and communicate the benefits of preventing child marriage in Indonesia.

## Declaration of Competing Interest

We declare no competing interests. The authors whose names are listed immediately below certify that they have no affiliations with or involvement in any organisation or entity with any financial interest or non-financial interest in the subject matter or materials discussed in this manuscript. Nicholas Goodwin has a commercial contract between UNICEF and Tulodo.

## Appendix Supplementary materials

Image, application 1

Image, application 2
