# Supplementary material for: One household, two worlds: Differences of perception towards child marriage among adolescent children and adults in Indonesia
Source: Lancet Reg Health West Pac. 2021 Feb 5;8:100103. doi: 10.1016/j.lanwpc.2021.100103 (PMC8315322; doi:10.1016/j.lanwpc.2021.100103)
Supplement: Supplementary file 1 [file mmc1.pdf]

STROBE Statement — Checklist of items that should be included in reports of ***cross-sectional studies***

	Item No	Recommendation
**Title and abstract**	1	*(a) Indicate the study's design with a commonly used term in the title or the abstract*<br><br>One Household, Two Worlds: Differences of Perception Towards Child Marriage Among Adolescent Children and Adults in Indonesia<br><br>Term "Differences of Perception" indicate cross sectional study
		(b) Provide in the abstract an informative and balanced summary of what was done and what was found<br><br>This cross sectional study was conducted in South Sulawesi Indonesia with a total of 1,004 respondents consisting of 500 parents with children aged 13-15 years and 504 adolescents aged 13-15 years joining the survey. This study found that around one out of four parents or adolescents had perceptions that support determinants of child marriage. A total of 25.8% of parents and 26.0% of adolescents agreed that a girl is ready for marriage once she starts menstruation. 25.6% of parents and 32.6% of adolescents agreed that girls aged over 18 who are not married are a burden to their families. Using the U-Mann Whitney Test, these differences were found to be significant.
**Introduction**		
Background/rationale	2	*Explain the scientific background and rationale for the investigation being reported*<br><br>Child marriage and menstrual health are two significant and interconnected health and human rights concerns in many low- and middle-income countries, including Indonesia. Around 45% of Indonesian girls feel unprepared for their first period, and only 16% of adolescent girls and 9% of adolescent boys know that a woman's fertile period is halfway between her periods. The child marriage burden remains too high in Indonesia, especially among female adolescents, despite positive long-term trends. Since 2008, there was a 3.85% decrease in the prevalence of child marriage from 14.67% in 2008 to 10.82% in 2019. The decline occurred in around three-quarters of provinces, mostly in rural areas. This continues a longer-term trend in the decline of child marriage in Indonesia - the risk of marrying before age 18 is less than half of what it was three decades ago. Based on 2017 data from the Village Data System (*Sistem Data Desa Kelurahan* = SDDK), from the 266,378 children in Bone District, the number of child marriage cases reached 2,635.
Objectives	3	*State specific objectives, including any prespecified hypotheses*<br><br>This study explores child marriage-related perceptions among parents and adolescents particularly perceptions related to the determinants of child marriage (e.g., socioeconomic factors, culture) including the perception of whether a girl is ready for marriage once she starts menstruating. This study also explores whether parents and adolescents have different perceptions of child marriage. Based on some studies, we found that parents' decision contributes to child marriage.
**Methods**		
Study design	4	*Present key elements of study design early in the paper*<br><br>This study used a cross-sectional design, with researchers making observations or measurements of variables at a certain time. The participants consisted of two groups: parents or caregivers in households with children aged 13-15 years and adolescents aged 13-15 years.
Setting	5	*Describe the setting, locations, and relevant dates, including periods of recruitment, exposure, follow-up, and data collection*<br><br>The study was conducted in six intervention villages and two control areas in Bone, South Sulawesi (Abbumpungeng, Cumpiga, Lamuru, Lilina Ajangale, Malimongeng, and Welado village as intervention villages, and Bontocani and Libureng village as the control areas). The data collection was conducted from 14 July to 4 August 2019 followed by the data analysis and report development.

Participants	6	*(a) Give the eligibility criteria, and the sources and methods of selection of participants*<br><br>The inclusion criteria for the study were (1) adolescents aged 13-15 years from 12 intervention schools in 6 sub-districts and 4 schools from control areas, and those dropping out of school/not continuing their education. For adolescents survey, we conducted the survey at school and visited those dropping out of school (2) parents with children aged 13-15 years in 6 intervention villages and 2 control villages. For the household survey, with the help of the village government, we mapped the households and determined which respondents were visited by using random techniques. We selected one house for every 10 houses, and if there were no families with adolescents aged 13-15 years within the 10 houses range, we visited the house next door until we found those with children aged 13-15 years, asked the households to join the study, and continued the data collection.
Variables	7	*Clearly define all outcomes, exposures, predictors, potential confounders, and effect modifiers. Give diagnostic criteria, if applicable*<br><br>This study will show different perceptions among parents and adolescents toward child marriage, its determinants, and its impact. The independent variables were demographics: gender, type of respondents (parent/caregiver vs adolescent), and location. The dependent variables in this study were the level of perception related to child marriage and menstrual health. This study also explored factors that influence parent/caregiver intention to arrange a marriage for their adolescents. This included adolescent intention and acceptance to marry early, as well as parent/caregiver efforts to provide education on health and relationships.
Data sources/ measurement	8*	*For each variable of interest, give sources of data and details of methods of assessment (measurement). Describe comparability of assessment methods if there is more than one group*<br><br>Prior to the data collection, we conducted enumerator training to ensure all enumerators have the same understanding on the research instruments. A pilot testing with 30 respondents was conducted to ensure that the research instruments used were easy to understand. Data collection was carried out by the two main researchers and assisted by eight enumerators. We have two groups (parents/caregiver groups and adolescents). All data was entered into the mWater Surveyor App on tablets. To prevent missing data, we set the mWater system so that only completed data can be submitted. For the accuracy data, the main researcher checked the data from enumerator user name on mWater Surveyor. We conducted a data cleaning method by the main researcher to ensure that all data has been submitted by enumerators into the mWater system.
Bias	9	*Describe any efforts to address potential sources of bias*<br><br>To reduce bias, we conducted pilot testing of the research instruments and increased the sample size. All protocols of the study have been reviewed and approved by the Ethics Committee from the Universitas Indonesia. During the data collection, enumerators were not allowed to help respondents answer the surveys when conducting interviews.
Study size	10	*Explain how the study size was arrived at*<br><br>We calculated the sample using Hulley et al's 2013 method, where N (sample) = 16 (standardised size effect). The standardised size effect to be used is 0.3, with a confidence interval of 95%, and power (β) of 80%. Using this formula, the sample for each group needed was 177 people. The total required sample for both boys and girls were 354 adolescents aged 13-15 years. Adding 10% to allow for problems, the total sample recruited was 389 adolescents, both in and out of school. The same sampling method and size for the adolescent survey was used for the parent/caregiver survey, with a total sample needed of 389 people. The total respondents joined the study was 1,004 people consisting of 500 parents with children aged 13-15 years and 504 adolescents aged 13-15 years.
Quantitative variables	11	*Explain how quantitative variables were handled in the analyses. If applicable, describe which groupings were chosen and why*

		To analyze demographic data, such as gender, the use of media, and technology, descriptive statistics were used. To answer the research question, inferential statistics were used. The questions of attitudes related to child marriage were explored as the dependent variables in this study were taken from the Acceptability Child Marriage Index (ACMI) developed by Plan International and standard questionnaire from UNICEF related to child marriage. The responses were arranged along a 7-level Likert scale consisting of: strongly disagree (1), disagree (2), slightly disagree (3), neither disagree nor agree (4), slightly agree (5), agree (6), and strongly agree (7). For further analyses, we grouped the responses above into two categories: (1) agree category (strongly agree/agree/slightly agree) and (2) disagree category (strongly disagree/disagree/slightly disagree).
Statistical methods	12	(*a*) Describe all statistical methods, including those used to control for confounding<br><br>For the demographic data, such as gender, the use of media, and technology, descriptive statistics, we used descriptive statistics such as analysis of frequency. For the inferential statistics, as the nature of data is ordinal, we used the U-Mann Whitney Test to analyze the differences in perception between parents/caregivers and adolescents. To analyze the association between attitudes and gender, type of respondents, and locations, we used Kendall's tau-b for Likert-type items and Pearson's correlation test for Likert scales.
		(b) Describe any methods used to examine subgroups and interactions<br><br>The subgroups in this study are adolescent groups and parent groups. To analyze the differences in perception between parents/caregivers and adolescents, we used the U-Mann Whitney Test. This study does not examine interactions.
		(c) Explain how missing data were addressed<br><br>This study has minimized the potential of missing data as we have set the submission data system on the mWater that data cannot be submitted when some data are missing.
		(d) If applicable, describe analytical methods taking account of sampling strategy<br><br>For the sampling strategy, we used a confidence interval of 95%, and power (β) of 80%. By using the Hulley et al, the sample for each group needed was 177 people. The total required sample for both boys and girls were 354 adolescents aged 13-15 years and same sample size for the households.
		(e) Describe any sensitivity analyses<br><br>We used Cronbach's alpha in the pilot testing and Pearson correlation
**Results**		
Participants	13*	*(a) Report numbers of individuals at each stage of study—eg numbers potentially eligible, examined for eligibility, confirmed eligible, included in the study, completing follow-up, and analysed*<br><br>A total of 500 respondents joined in the parents/caregivers survey and 504 adolescents aged 13-15 years. For descriptive analysis, a total of 1004 data eligibility for analysis. For comparative analysis of perceptions, we calculated the number of data based on their answers for each statement. The average number of data analysed from two groups (parents/caregiver : 498 data and adolescents : 473 data)
		(b) Give reasons for non-participation at each stage<br><br>One of reasons for non-participation in the analysis stage was respondents answered "neither agree nor disagree" in the Likert scale questions. This response was not counted in the Mann Whitney, Kendall's Tau-b and Pearson correlation.
		(c) Consider use of a flow diagram

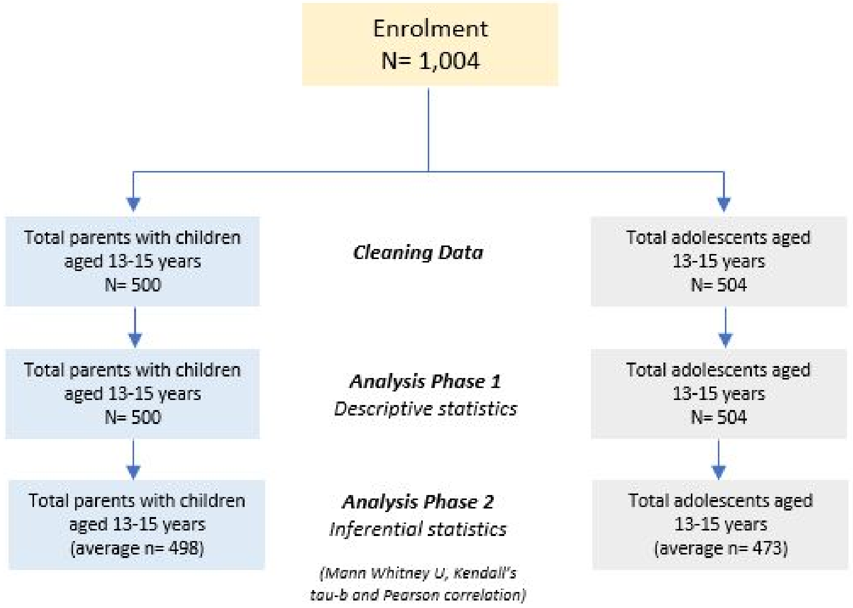

Descriptive data	14*	*(a) Give characteristics of study participants (eg demographic, clinical, social) and information on exposures and potential confounders*<br><br>A total of 500 respondents joined the parents/caregivers survey, chosen randomly in 6 intervention areas (Ajangngale, Awangpone, Cina, Salomekko, Tellusiattingnge and Ulaweng) and two control subdistricts (Libureng and Bontocani). 64.4% (n=322) were from intervention areas while 35.6% (n=178) were from the control. 416 respondents were female (83.2%), while 84 were male (16.8%). Most respondents were mothers or primary caregivers of adolescents-aged 13-15 years (72.2%, n=361) whilst 20% (n=100) were heads of households. Out of 361 primary caregivers, 86.3% (n=359) were female respondents whilst 2.4% (n=2) were male. 38.4% (n=192) had completed elementary school, 21.4% (n=107) completed junior high school, and 17% (n=85) graduated from senior high school. For heads of household, 41% (n=205) completed elementary school, 17.4% (n=87) graduated from junior high school and 16.2% (n=81) graduated from senior high school.<br><br>A total of 504 adolescents aged 13-15 years joined the study, consisting of 24 out-of-school and 480 students from 16 schools in 8 sub-districts in Bone (6 intervention sub-districts and 2 control). There were 254 boys (50.4%), and 250 girls (49.6%). Most respondents were in grade IX (224 respondents, 44.4%), the rest were in grade VII and VIII, with most (38.1%) aged 13 years (n=192).
		(b) Indicate number of participants with missing data for each variable of interest<br><br>This study has minimized the potential of missing data as we have set the submission data system on the mWater that data cannot be submitted when some data are missing.
Outcome data	15*	*Report numbers of outcome events or summary measures*<br><br>A statistically significant difference in terms of responses to the statement, “a girl is ready for marriage once she starts menstruating” between the type of respondents, p = 0.034, with a mean rank 520.69 for parents and 484.45 for adolescents. In terms of the statement, “Girls over 18 who are not married are a burden to their families”, there was a significant difference between the type of respondents, p = 0.002, with a mean rank of 475.71 for parents and 529.80 for adolescents. The other significant differences were found in terms of the statements, “Boys over 18 who are not married are a burden to their families” (p = 0.009), “Most adolescent girls prefer to marry before 18” (p=0.011), and “parents expect adolescent girls to get married before the age of 18 years” (p = 0.002).

		A statistically significant association in terms of the statement, "Most adolescent girls prefer to marry before 18 years by type of respondents between parents and adolescents (p=0.011; r=0.072).<br><br>A statistically significant association in terms of responses to the statement, "physical changes in appearance is a sign that a girl is ready for marriage" by type of respondents between parents and adolescents (p=0.000; r=0.143). There was a statistically significant association in terms of the statement, "parents expect adolescent girls to get married before the age of 18 years" by gender between female and male (p=0.038; r=0.066); by location between intervention and control (p=0.000; r=0.119). There was a statistically significant association in terms of the statement "Parents would look down on adolescent girls if they get pregnant before they get married" by type of respondents between parents and adolescents (p=0.000; r=0.122).
Main results	16	*(a) Give unadjusted estimates and, if applicable, confounder-adjusted estimates and their precision (eg, 95% confidence interval). Make clear which confounders were adjusted for and why they were included*<br><br>When we analyzed the data, we didn't find any significant confounding variables. To ensure that the resulting data are valid, we use a 95% confidence interval for Mann Whitney test, Kendall's Tau-b test, and Pearson correlation test.
		(b) Report category boundaries when continuous variables were categorized<br><br>For the Acceptability Child Marriage Index (ACMI) variables (the 7 level of Likert scale), we categorize the variables based on the answers:<br>● Respondent's answer Strongly Agree, Agree, Slightly Agree were categorized into Agree.<br>● Respondent's answer Netral (Neither disagree nor agree (neutral) were not included for comparative analysis<br>● Respondent's Slightly Disagree, Disagree, Strongly Disagree were categorized into Disagree.
		(c) If relevant, consider translating estimates of relative risk into absolute risk for a meaningful time period<br><br>We didn't calculate relative risk for this study. This study calculates the difference in perceptions between parents / caregivers and adolescents.
Other analyses	17	Report other analyses done—eg analyses of subgroups and interactions, and sensitivity analyses<br><br>We didn't calculate interaction for each subgroup for this study. For sensitivity analyses, we used Cronbach's alpha to know reliability and Pearson correlation to know validity of data in pilot tests.
**Discussion**		
Key results	18	Summarise key results with reference to study objectives:<br>● *Support for child marriage still high because of pride.* This study found that 29.6% of parents and 33.4% of adolescents strongly agreed/agreed/slightly agreed that marrying girls can help protect family honours /reputation. The perception of the benefits of child marriage in terms of protecting family honours /reputation was significantly higher among adolescents than those of parents.

		● *Support for child marriage still high because of economic factors.* Marriage is sometimes used as a means to rise out of poverty and into higher social status. Around 23.2% of parents and 26.0% of adolescents strongly agreed/agreed/slightly agreed that marrying a girl young can help resolve financial problems in the family; 25.6% of parents and 32.6% of adolescents strongly agreed/agreed/slightly agreed that girls over 18 who are not married are a burden to their families. Different perceptions related to economic problems can lead to stronger adolescent perceptions towards the benefits of child marriage than parents. Some adolescents see aspects of marriage as helping to improve the economic life of the married couple or child.<br>● *Child marriage and unwanted pregnancy.* Around 43.8% of parents and 22.6% of adolescents agreed - also 6.8% of parents and 13.7% of adolescents strongly agreed - that parents would look down on adolescent girls if they became pregnant before they marry. There are differences in perception related to unwanted pregnancy, with stronger support for child marriage as a solution from parents than adolescents.<br>● *Menstruation, puberty and child marriage.* Around 25.8% of parents and 26.0% of adolescents strongly agreed/agreed and slightly agreed that a girl is ready for marriage once she starts menstruating, 26.0% of parents and 26.6% of adolescents agreed to physical changes in appearance are a sign that a girl is ready for marriage. Perceptions about menstruation and puberty as a contributor to child marriage are stronger in adolescents than parents.<br>● *Child marriage and sexual harassment.* Around 25.2% of parents and 29.4% of adolescents agree that early marriage of girls can help prevent sexual violence, assault, and harassment. The perception of child marriage as protection against sexual harassment is stronger in adolescents than in parents.
Limitations	19	*Discuss limitations of the study, taking into account sources of potential bias or imprecision. Discuss both direction and magnitude of any potential bias*<br><br>The limitations of this study include the nature of the cross sectional design study that cannot explore what causes the different perceptions of the child marriage between adolescents and parents and its analogous explanations/causal inference. The use of a 7-level Likert scale might also lead to respondents' confusion over the questions.
Interpretation	20	*Give a cautious overall interpretation of results considering objectives, limitations, multiplicity of analyses, results from similar studies, and other relevant evidence*<br><br>The prevalence of child marriage in Indonesia is still high (10.82%). A large proportion of females are still entering into child marriage placing young mothers and their adolescents at significant risk. Several studies explore factors underlying child marriage practices include limited economic and educational opportunities, cultural norms, poverty, and parental concerns with safeguarding their daughters' virginity. Parents are primary decision-makers in child marriage. The lack of power in decision making among girls also means parents tend to make decisions for their daughters about when and whom to marry but they do not fully bear the impact of those decisions.<br><br>Overall, the perceptions of parents and their adolescent children do not greatly differ, with some notable exceptions. Positive perceptions towards the benefits of child marriage are still prevalent among both parents and adolescents. This indicates that social norms supporting child marriage are still strong among these groups.This study adds value as it is the first in Indonesia to explore perceptions among parents and adolescents related to the determinants of child marriage, e.g socio-economic, education, and culture. This study is one of the first to document an intervention that links child marriage with menstrual health in Indonesia. The research also adds value to studies of making decision processes within families and communities, particularly on health and social issues.<br><br>Previous evidence has established an understanding of the determinants of, and pathways to, child marriage in various contexts. Some of these examine the attitudes of parents and young people, few compare statistically significant cohorts of parents and their adolescent children in the same area, with none from Indonesia. Given the

		primacy of this relationship, our study explores perceptions of child marriage and its determinants between adolescents and parents in Bone, South Sulawesi. By exploring the differences and similarities between these two groups, we have produced findings that are being utilised to design tailored interventions and services to prevent child marriage.
Generalisability	21	*Discuss the generalisability (external validity) of the study results*<br><br>This study represents adolescents aged 13-15 years and parents with children aged 13-15 years in Bone, South Sulawesi. This study may provide same results when applied in other areas around Bone Regency (e.g., neighbouring regencies/districts) as they have similar ethnicity. However there is a need to conduct the further study to conclude this as the results may differ from those enrolled in the study.
**Other information**		
Funding	22	*Give the source of funding and the role of the funders for the present study and, if applicable, for the original study on which the present article is based*<br><br>This study obtained funding from UNICEF and UNFPA in partnership with the Indonesian Government, and the Canadian Government through the BERANI (Better Reproductive Health and Rights for All in Indonesia) program for the baseline study in Bone, South Sulawesi.

*Give information separately for exposed and unexposed groups.

Note: An Explanation and Elaboration article discusses each checklist item and gives methodological background and published examples of transparent reporting. The STROBE checklist is best used in conjunction with this article (freely available on the Web sites of PLoS Medicine at http://www.plosmedicine.org/, Annals of Internal Medicine at http://www.annals.org/, and Epidemiology at http://www.epidem.com/). Information on the STROBE Initiative is available at www.strobe-statement.org.
