# Supplementary material for: One household, two worlds: Differences of perception towards child marriage among adolescent children and adults in Indonesia
Source: Lancet Reg Health West Pac. 2021 Feb 5;8:100103. doi: 10.1016/j.lanwpc.2021.100103 (PMC8315322; doi:10.1016/j.lanwpc.2021.100103)
Supplement: Supplementary file 2 [file mmc2.pdf]

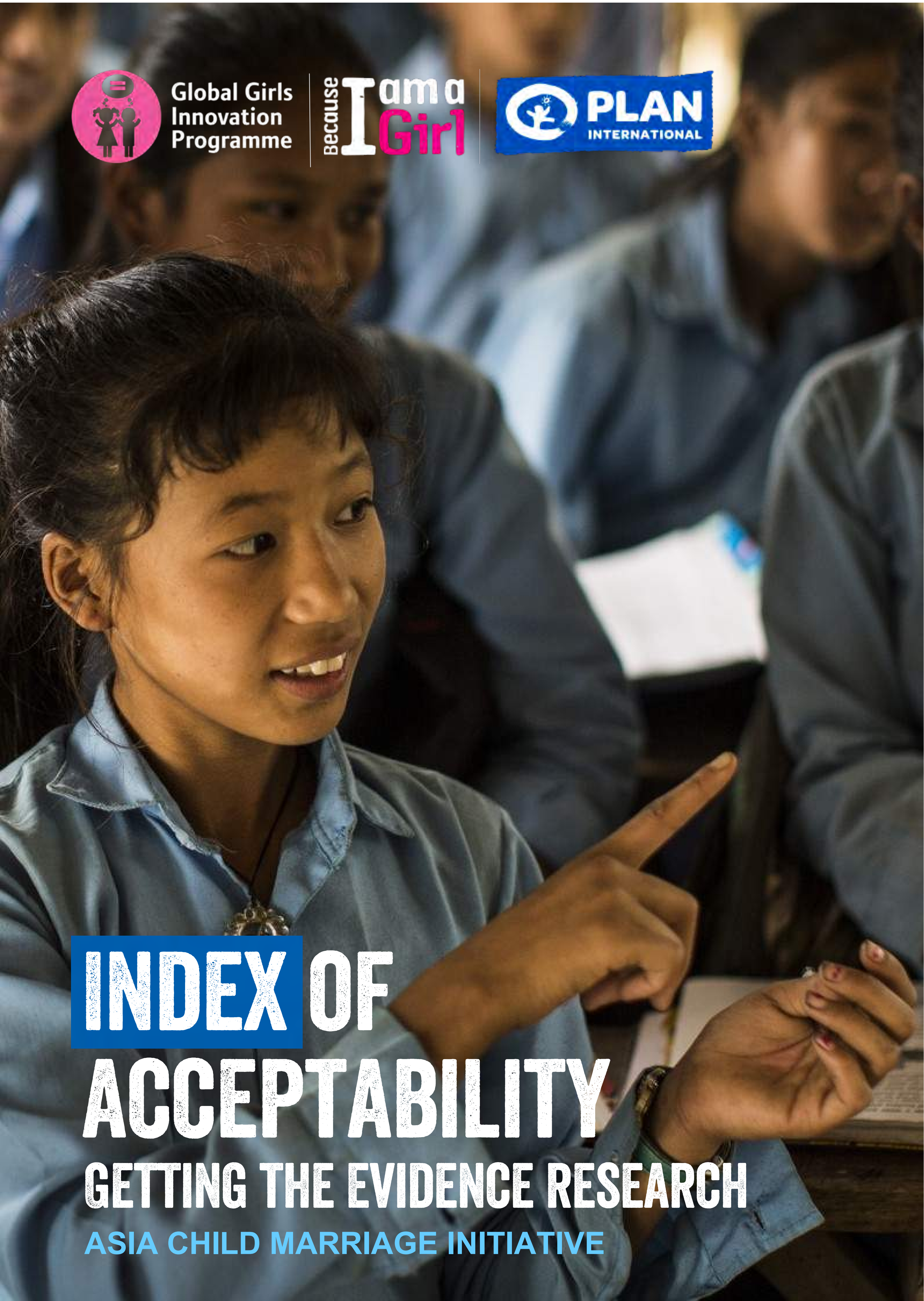

Global Girls Innovation Programme

Because I am a Girl

PLAN INTERNATIONAL

INDEX OF ACCEPTABILITY

GETTING THE EVIDENCE RESEARCH

ASIA CHILD MARRIAGE INITIATIVE

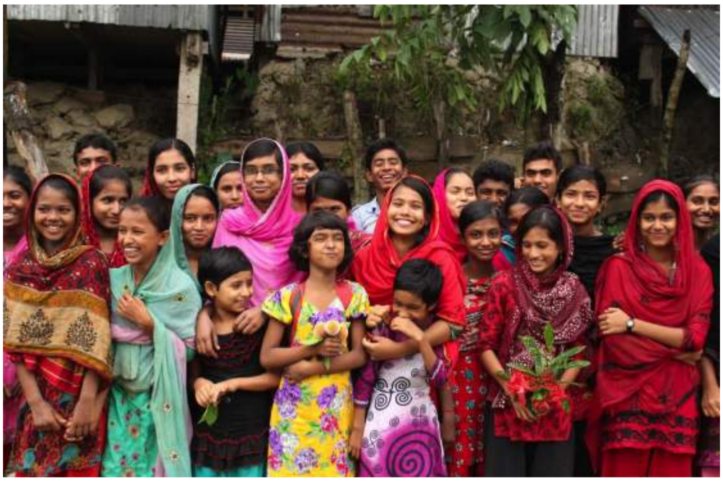

As part of the Year 1 of the ACMI Research, the CCLC has developed an index designed to 'score' communities according to the presence/absence of indicators associated with norms and attitudes about when a girl's marriage is necessary, desirable, acceptable or unacceptable.

1. WHY AN INDEX?

The first year of ACMI research revealed that practices and attitudes concerning child marriage are heavily influenced and shaped by environmental factors: such as economic deprivation, gender inequality, dependency and violence, and lack of education and employment opportunities. Thus, programming aimed at eradicating child marriage must focus on addressing these underlying drivers (environmental factors) that shape attitudes and practices.

The index has been developed with this in mind: in order to help Plan design programme interventions that target these 'drivers' more effectively and track their impact.

2. ASSUMPTIONS

The index has been designed in recognition of an increasing body of evidence and theory that suggests that the practice and prevalence of child marriage is (partially) influenced by cultural norms that create and reinforce ideas, and shape expectations, about the *acceptability* of child marriage, both in general and within particular circumstances.

These norms and attitudes are themselves influenced by structural factors, such as legal and policy systems, inequalities in access to resources, benefits and services, and the distribution of social, economic and political power more broadly.

> For example, a father who is very poor and has many children whom he struggles to clothe, feed, and educate, is more likely to find child marriage acceptable than a rich father with few children.

Because dominant norms and attitudes concerning child marriage are (partially) determined by social and material conditions (structural factors), changes in conditions/ factors (e.g. improvements in education, reduction of poverty etc.) are likely to have an impact on these ideas and support the emergence of norms that are prohibitive rather than supportive of the practice of child marriage.

3. PURPOSE OF THE INDEX

The index may be used by Plan to:

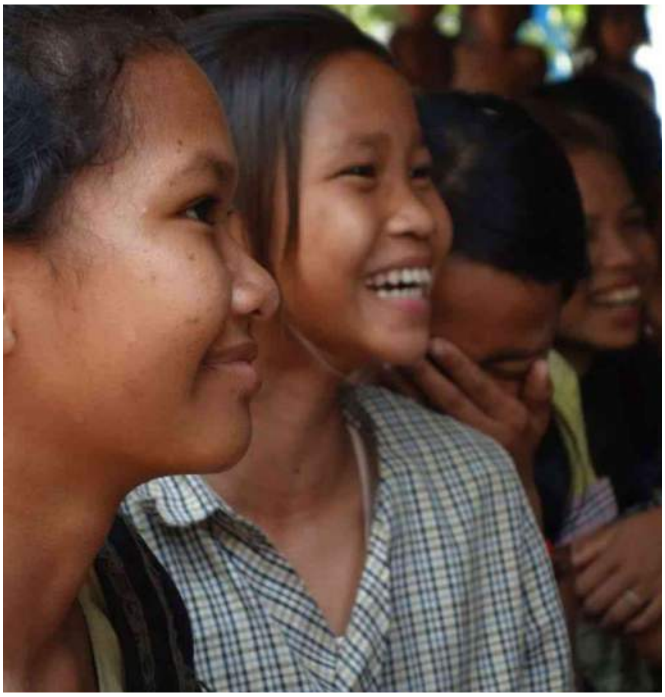

1. Assess levels of 'risk' of child marriage acceptability in a community (i.e. before designing a programme intervention), according to the presence of structural drivers associated with higher levels of child marriage (acceptability)

2. Determine programming interventions and anticipate how likely a community is to be accepting of the practice of child marriage

3. Track and measure changes in both structural drivers associated with child marriage acceptability, and levels of (predicted) community acceptability over time. This expected rate of change, depending on interventions, lends itself to periodic evaluations or studies (every 3-5 years), rather than routine monitoring which would be too frequent.

4. LIMITATIONS

The index is a tool for anticipating how likely a community is to be accepting of the practice of child marriage, based on a range of environment factors. The reason that this is useful is that it can provide information about the potential impact, over time, of Plan's programming across a range of different thematic areas on child marriage acceptability.

The index cannot be used for any other purpose. It is important to recognize that it does not provide information about *actual* levels of child marriage acceptability. Neither does it provide information about actual *rates* of child marriage in practice.

5. INDICATORS

The index is comprised of a set of indicators (and associated variables) that signal (reveal) the presence of structural/ environmental factors associated with child marriage (acceptability). These were identified during the Year 1 research in Bangladesh, Indonesia and Pakistan, through analysis of survey data collected. They include:

1. Access to sexual and reproductive health/ Fertility
2. Financial Security
3. Education rates
4. Legal Frameworks
5. Dowry
6. Sexual and Gender Based Violence

Each community was assigned a 'value' for each indicator based on the relative presence of that indicator on a scale from 0-30.

Indicators were selected on the basis of their strength of association with child marriage acceptability (as demonstrated by data collected during year 1 of the ACMI research). Structural factors that do not relate to areas of Plan programming were excluded (e.g. level of rurality).

6. VARIABLES

Each indicator is broken down into a series of 'variables' which provide specific information in relation to the data that should be collected in relation to each indicator.

Weighting

Each variable has been assigned a weighting according to its relative influence on attitudes that are either accepting or unaccepting of child marriage.

The weight of indicators was determined through analysis of the qualitative and quantitative data collected during Year 1 of the ACMI research. Where there was found to be a relatively strong relationship/association between a particular indicator and ideas accepting of child marriage, the indicator was given a high weighting (e.g. girls' education). Where there was found to be a weak association between a particular indicator and ideas which are accepting of child marriage, the indicator was given a low weighting (e.g. legal identity).

Quantitative methods for calculating weighting

Each individual surveyed during the research was assigned a 'child marriage acceptability score' based on their responses to survey questions, designed to collect data on dominant norms and attitudes relating to (child) marriage.

Participants were read a statement (such as "there are advantages to marriage under 18 years") and then asked to express their level of agreement on a likert scale with 7 variables: strongly agree; agree; slightly agree; neutral; slightly disagree; disagree; strongly disagree. Statements comprised those both accepting and unaccepting of child marriage practices. For each response the lowest score (1) is assigned to a response which reflects low acceptability of child marriage, and the highest score (7) represents high levels of acceptability of child marriage. The responses were aggregated to assign each respondent an 'acceptability score' on a scale of 23 – 161.

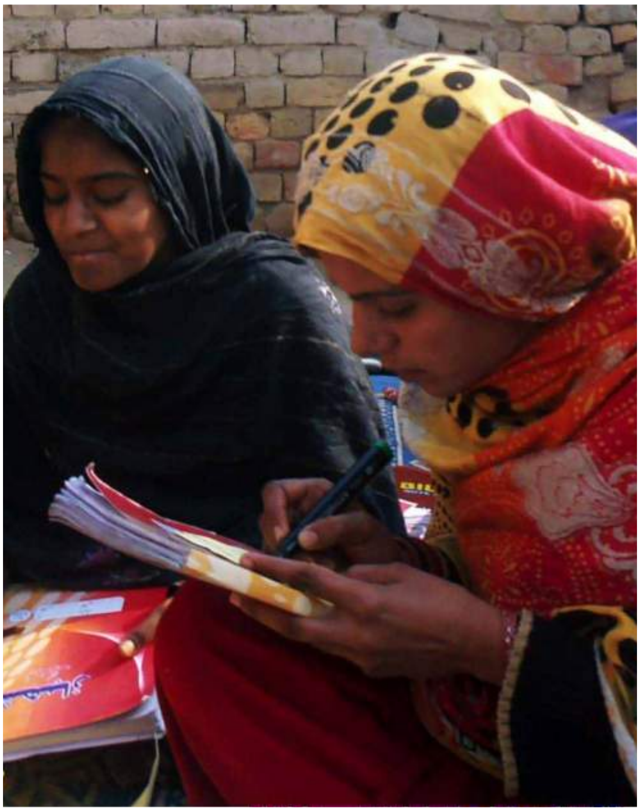

Table 1: List of statements and how they were scored

The acceptability score was been developed based on the recognition that attitudes about child marriage are complex, contextual, fluid and non-homogenous. Because of this the acceptability score included a set of 23 different questions to determine attitudes and perceptions that relate to various elements of acceptability.

No.	Perception	Score
**1.**	Ideal age of marriage for girls.	*(1) = >25; (2) = 23-25; (3) = 21-22; (4) = 19-20; (5) = 18; (6) = 16-17; (7) = <16*
**2.**	Disparity in ideal age of marriage for girls compared to boys.	*1) = 0 years; (2) = 1 year; (3) = 2 years; (4) = 3 years; (5) = 4 years; (6) = 5 years; (7) = <5 years*
**3.**	Lowest acceptable age of marriage for girls.	*(1) = 18 years or more; (2) = 17; (3) = 16; (4) = 15; (5) = 14; (6) = 13; (7) = 12 years or below*
**4.**	Disparity in lowest acceptable age of marriage for girls compared to boys.	*1) = 0 years; (2) = 1 year; (3) = 2 years; (4) = 3 years; (5) = 4 years; (6) = 5 years; (7) = <5 years*
**5.**	Highest acceptable age of marriage for girls.	*(1) = no upper limit; (2) = >40; (3) = 30-40; (4) = 25-29; (5) = 21-24; (6) = 19-20; (7) = 18 years or lower*
**6.**	Disparity in highest acceptable age of marriage for girls compared to boys.	*1) = 0 years; (2) = 1 year; (3) = 2 years; (4) = 3 years; (5) = 4 years; (6) = 5 years; (7) = <5 years*
**7.**	A girl is ready for marriage once she starts menstruating	*(1) = strongly disagree; (2) = disagree; (3) = slightly disagree; (4) = neutral; (5) = slightly agree; (6) = agree; (7) = strongly agree.*
**8.**	There are advantages to marriage of girls under 18 years	*(1) = strongly disagree; (2) = disagree; (3) = slightly disagree; (4) = neutral; (5) = slightly agree; (6) = agree; (7) = strongly agree.*
**9.**	There are disadvantages for girls getting married under 18 years.	*(1) = strongly disagree; (2) = disagree; (3) = slightly disagree; (4) = neutral; (5) = slightly agree; (6) = agree; (7) = strongly agree.*
**10.**	Marrying girls young can help protect family honour/ reputation.	*(1) = strongly disagree; (2) = disagree; (3) = slightly disagree; (4) = neutral; (5) = slightly agree; (6) = agree; (7) = strongly agree.*
**11.**	Girls who give birth between 15-18 years are more likely to have a healthy pregnancy/ baby (compared to girls over 18).	*(1) = strongly disagree; (2) = disagree; (3) = slightly disagree; (4) = neutral; (5) = slightly agree; (6) = agree; (7) = strongly agree.*
**12.**	Marrying girls young can help resolve financial problems in the family.	*(1) = strongly disagree; (2) = disagree; (3) = slightly disagree; (4) = neutral; (5) = slightly agree; (6) = agree; (7) = strongly agree.*
**13.**	Marrying girls young can help provide them security.	*(1) = strongly disagree; (2) = disagree; (3) = slightly disagree; (4) = neutral; (5) = slightly agree; (6) = agree; (7) = strongly agree.*
**14.**	Early marriage of girls can help prevent sexual violence, assault and harassment.	*(1) = strongly disagree; (2) = disagree; (3) = slightly disagree; (4) = neutral; (5) = slightly agree; (6) = agree; (7) = strongly agree.*
**15.**	Early marriage of boys can help prevent perpetration of sexual violence, assault and harassment.	*(1) = strongly disagree; (2) = disagree; (3) = slightly disagree; (4) = neutral; (5) = slightly agree; (6) = agree; (7) = strongly agree.*
**16.**	Marrying under 18 years is likely to have a negative impact on a girls' education.	*(1) = strongly agree; (2) = agree; (3) = slightly agree; (4) = neutral; (5) = slightly disagree; (6) = disagree; (7) = strongly disagree.*

**17.**	Marrying a girl young is preferable because younger brides are more obedient and respectful of their husbands.	*(1) = strongly disagree; (2) = disagree; (3) = slightly disagree; (4) = neutral; (5) = slightly agree; (6) = agree; (7) = strongly agree.*
**18.**	Even if a girl does not want to be married, she should honour the decisions/ wishes of her family	*(1) = strongly disagree; (2) = disagree; (3) = slightly disagree; (4) = neutral; (5) = slightly agree; (6) = agree; (7) = strongly agree.*
**19.**	Younger brides require a lower dowry than older brides.	*(1) = strongly disagree; (2) = disagree; (3) = slightly disagree; (4) = neutral; (5) = slightly agree; (6) = agree; (7) = strongly agree.*
**20.**	A girl should never be forced or compelled into marriage.	*(1) = strongly agree; (2) = agree; (3) = slightly agree; (4) = neutral; (5) = slightly disagree; (6) = disagree; (7) = strongly disagree.*
**21.**	It is sometimes ok to beat or punish a girl when she dishonours her family.	*(1) = strongly disagree; (2) = disagree; (3) = slightly disagree; (4) = neutral; (5) = slightly agree; (6) = agree; (7) = strongly agree.*
**22.**	A wife should be subservient to her husband.	*(1) = strongly disagree; (2) = disagree; (3) = slightly disagree; (4) = neutral; (5) = slightly agree; (6) = agree; (7) = strongly agree.*
**23.**	Men should be the heads of their household.	*(1) = strongly disagree; (2) = disagree; (3) = slightly disagree; (4) = neutral; (5) = slightly agree; (6) = agree; (7) = strongly agree.*

After calculating these acceptability scores the team ran a series of statistical tests, including bivariate correlation, annova analysis of variance and regression analysis to test the existence and strength of associations between structural factors (the variables in the index) and their child marriage acceptability scores.

The strength of these associations was triangulated in light of the qualitative data. Subsequent triangulation, weighting values were assigned to each indicator based on their relative importance for influencing child marriage acceptability.

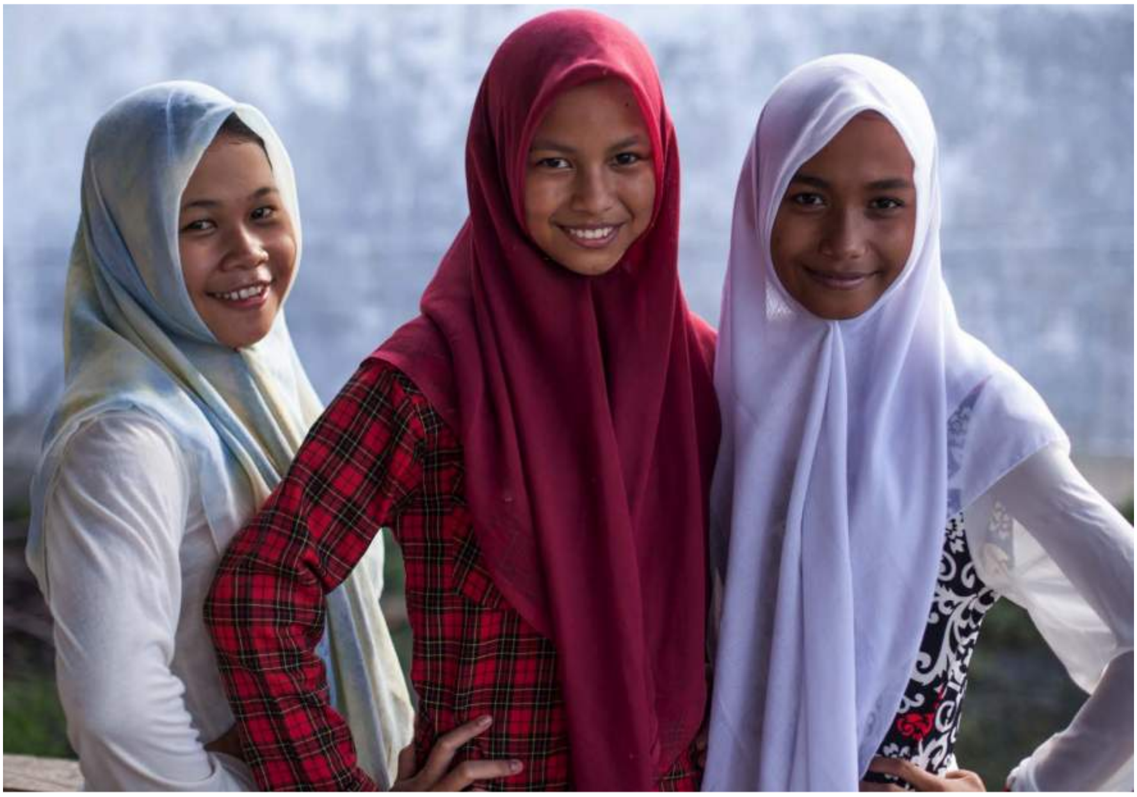

7. CHILD MARRIAGE ACCEPTABILITY INDEX FOR PROGRAMMING

Indicator	Relevant variables on which to collect data		Indicator value/scoring guide (0 – 10)	Total Score	Variable weighting (Scale of 0-1)	Associated Programme Target(s)
Access to Sexual and Reproductive Health	1. **No. of pregnancies (married women only)**		(8+)=5 (5-7)=4 (4)=3 (3)=2 (2-1)=0	Max possible score = 5	0.21	
	2. **No. of children (married men and women)**		(8+)=5 (5-7)=4 (4)=3 (3)=2 (1-2)=0	Max possible score = 5	0.21	
	3. **Age of first pregnancy (married women only)**:		(<18 years)=5 (18-19 years)=4 (20-21 years)=3 (22-24 years)=2 (25-29 years)=1 (30+ Years)=0	Max possible score = 5	0.21	
	4. **Size of household of (adult men only):**		(10+)=5 (8-9)=4 (7)=3 (6)=2 (5)=1 (2-4)=0	Max possible score = 5	0.21	
	5. **Reported use of modern contraception (married women only)**		(Doesn't use contraception)=5 (uses contraception)=0	Max possible score = 5	0.21	
	6. **Ability to identify modern contraception available in their community (women/ girls only)**		(Not able to identify any type)=5 (able to identify one type)=3 (able to identify multiple types)=0	Max possible score = 5	0.21	
Household financial security	1. **Average monthly income (adult men)**		(Income is in top 10% of national average)=0<br>(4th quintile/ top 60-80%)=1<br>(Middle/3rd quintile/ 40-60%)=2<br>(2nd quintile/ bottom 20-40%)=3<br>(1st quintile bottom 20%)=4<br>(Living below the poverty line)=5	Max possible score = 5	0.35	
	2. **Average monthly income (adult women)**		(Income is in top 10% of national average)=0<br>(4th quintile/ top 60-80%)=1<br>(Middle/3rd quintile/ 40-60%)=2<br>(2nd quintile/ bottom 20-40%)=3<br>(1st quintile bottom 20%)=4<br>(living below the poverty line)=5	Max possible score = 5	0.17	
	3. **How often does the household have**	**Sufficient food so as not to go hungry**	(Always)=0 (Most of the time)=2 (Sometimes)=3 (Never)=4	Max possible score = 4	0.26	
		**Clothes**	(Always)=0 (Most of the time)=2 (Sometimes)=3 (Never)=4	Max possible score = 4	0.26	

	**enough of the following items (all)**	**Regular Medicines**	(Always)=0 (Most of the time)=2 (Sometimes)=3 (Never)=4	Max possible score = 4	0.26	
		**School items (uniforms/ books etc.**	(Always)=0 (Most of the time)=2 (Sometimes)=3 (Never)=4	Max possible score = 4	0.26	
		**Extra items (gifts, travel etc.)**	(Always)=0 (Most of the time)=2 (Sometimes)=3 (Never)=4	Max possible score = 4	0.26	
Education	**1. Type of schooling (all)**		(Formal/ mainstream education)=0 (Basic education/ Madrassa)=3 (No education)=5	Max possible score = 5	0.54	
	**2. Years of school completed (all)**		(12+ years)=0 (9-12 years)=1 (6-8 years)=3 (1-5 years)=4 (0 years)=5	Max possible score = 5	0.37	
	3. **Are girls in school (girls aged 6-18 years only)**		(Yes)=0 (No)=5	Max possible score = 5	0.95	
	4. **Are boys in school? (boys aged 6-18 years only)**		(Yes)=0 (No)=5	Max possible score = 5	0.41	
	5. **Mother's Education (all)**		(Formal/ mainstream education)=0 (Basic education/ Madrassa)=3 (No education)=5	Max possible score = 5	0.06	
	6. **Father's Education (all)**		Formal/ mainstream education)=0 (Basic education/ Madrassa)=3 (No education)=5	Max possible score = 5	0.14	
Legal Frameworks	1. **Correct knowledge of (existing) law which prohibits child marriage (all)**		(There is no law)=10 (unable to correctly identify law)=10 (Able to identify an existing, protective law)=0	Max possible score = 10	0.38	
	2. **Did the participant register their marriage? (married respondents only)**		(No)=10 (Yes)=0	Max possible score = 10	0.01	
	3. **Does the participant possess and identity document? (all)**		(No)=10 (Yes)=0	Max possible score = 10	0.01	
Dowry	1. **Do marriages in the community involve the exchange/ agreement regarding dowry? (all)**		(No)=0 (Yes)=10	Max possible score = 10	0.15	
	2. **Do younger brides require a lower dowry price? (Views of men/boys)**		(Strongly agree)=10 (Agree)=8 (Neutral)=5 (Disagree)=2 (Strongly Disagree)=0 (Not applicable/ no dowry)=0	Max possible score = 10	0.37	

	3. **Do younger brides require a lower dowry price? (Views of women/girls)**	(Strongly agree)=10 (Agree)=8 (Neutral)=5 (Disagree)=2 (Strongly Disagree)=0 (Not applicable/ no dowry)=0	Max possible score = 10	0.26	
SGBV	1. **Acceptance of sexual violence against women/ girls and male dominance/ control within marriage (all)**	(Strongly agree)=10 (Agree)=8 (Neutral)=5 (Disagree)=2 (Strongly Disagree)=0	Max possible score = 10	0.39	
	2. **Cultural/social belief that early marriage of girls prevents sexual harassment/ violence (views of men/boys)**	(Strongly agree)=10 (Agree)=8 (Neutral)=5 (Disagree)=2 (Strongly Disagree)=0	Max possible score = 10	0.99	
	3. **Cultural/social belief that early marriage of girls prevents sexual harassment/ violence (views of men/boys)**	(Strongly agree)=10 (Agree)=8 (Neutral)=5 (Disagree)=2 (Strongly Disagree)=0	Max possible score = 10	0.64	

Plan International
Regional Office in Asia
14th floor, 253 Sukhumvit 21 Road (Asok)
Klongtoey Nua, Wattana, Bangkok
10110 Thailand
www.plan-international.org/asia

About Plan International

Plan International is an independent global child rights organisation committed to supporting vulnerable and marginalised children and their communities to be free from poverty. By actively connecting committed people with powerful ideas, we work together to make positive, deep-rooted and lasting changes in children and young people's lives . years, we have supported girls and boys and their communities around the world to gain the skills, knowledge and confidence they need to claim their rights, free themselves from poverty and live positive fulfilling lives.
